# Supplementary material for: Cloud BioLinux: pre-configured and on-demand bioinformatics computing for the genomics community
Source: BMC Bioinformatics. 2012 Mar 19;13:42. doi: 10.1186/1471-2105-13-42 (PMC3372431; doi:10.1186/1471-2105-13-42)
Supplement: Additional file 1 — Supplementary 1 Cloud BioLinux software documentation in the form of a mini, self-contained website. Users need to download and uncompress the .zip file, and open through a web browser the "index.html" file available on the main directory. (ZIP 1823 kb). [file 1471-2105-13-42-S1.ZIP › Cloud-BioLinux-Package-Documentation/Edit.html]

Bio-Linux Software Documentation Pages

Back to search form

### Edit

|  |  |
| --- | --- |
| Category | Applications |
| Edit | splitsource    sizeseq    seqretsetall    nthseqset    notab    nospace    nohtml    aligncopypair    yank    vectorstrip    union    trimspace    trimseq    trimest    splitter    skipseq    skipredundant    seqrettype    seqretsplit    seqretset    seqretall    seqret    revseq    pasteseq    origunion    origsplitter    nthseq    notseq    noreturn    newseq    maskseq    maskfeat    maskambigprot    maskambignuc    makeprotseq    makenucseq    listor    featreport    featcopy    extractseq    extractfeat    extractalign    entret    descseq    degapseq    cutseq    codcopy    biosed    aligncopy |
| SubCategory | Applications |
